# From Nature to Strength: A Proof-of-Concept Study Integrating a Nature-Based Intervention with Virtually Supported Resistance Training in Young Men

**DOI:** 10.3390/healthcare14070937

**Published:** 2026-04-03

**Authors:** Alfred S. Y. Lee, Bradley A. Rudner, Ryan E. Rhodes, Nevin J. Harper

**Affiliations:** 1 Behavioural Medicine Laboratory, School of Exercise Science, Physical and Health Education, University of Victoria, Victoria, BC V8W 3N4, Canada; rhodes@uvic.ca; 2 School of Exercise Science, Physical and Health Education, University of Victoria, Victoria, BC V8W 2Y2, Canada; bradrudner@uvic.ca (B.A.R.); njharper@uvic.ca (N.J.H.)

**Keywords:** physical activities, mental health, self-determination theory, male, ORBIT model

## Abstract

**Background:** Young men experience substantial mental health and mortality-related risks, yet they often do not engage in conventional health promotion programs. This highlights the need for gender-specifc interventions that are acceptable, engaging, and feasible for young men. **Purpose of Research:** Guided by self-determination theory, this single-group proof-of-concept study evaluated the feasibility and acceptability of a dual-component intervention combining an in-person nature-based intervention (NBI; two days of group activities and guided reflection in a forested park) and a subsequent virtually supported resistance training (RT) program for young men and explored secondary, exploratory pre- to post-changes in depressive and anxiety symptoms. **Methods:** Eight men aged 18–34 not meeting RT recommendations (i.e., <2 sessions/week) completed a two-day, in-person NBI followed by six weeks of virtually supported RT with weekly group check-ins. Primary feasibility outcomes were satisfaction and qualitative acceptability for NBI/RT, recruitment, retention, and adherence. Secondary, exploratory quantitative outcomes were pre- to post-changes in depressive and anxiety symptom scores. Brief semi-structured exit interviews were conducted at the study end and audio-recorded for analysis. **Results:** Satisfaction met a priori thresholds for both components (NBI = 3.4/4; RT = 4.3/5; criteria ≥ 3.0 and ≥ 3.5). Recruitment was 46% and retention 100%, exceeding the 42% and 80% criteria, respectively. Exit interview themes highlighted guided learning, accountability, and feeling more connected to nature as acceptability drivers, with the scheduling burden noted but manageable. Depressive and anxiety symptom scores were lower post-intervention. **Conclusions:** Challenges in recruitment, group dynamics, and participant selection require refinement before the next phase; however, high satisfaction with both the NBI and RT segments, together with improvements in anxiety and depression symptom scores, supports progressing to a feasibility trial once these enhancements are in place.

## 1. Introduction

Young adult males are facing growing challenges across multiple domains, including academics, mental health, addiction, suicide, and all-cause mortality [1,2,3]. While historical gender disparities in education once disadvantaged women, recent trends show young women significantly outpacing men in academic achievement and university attendance [4,5]. Recent Global Burden of Disease (GBD) analyses provide updated, age- and sex-specific estimates of mortality and years of life lost and indicate substantial sex differences in mortality-driven disease burden, including large gaps for road injuries and other external causes [6,7]. In young men, external causes (e.g., transport injuries, violence), substance use, and suicide remain major contributors to mortality burden [8,9]. These troubling estimates point to a need for gender-sensitive health promotion strategies [10]. Although overt chronic disease is uncommon in this age group, cardiometabolic risk factors begin to consolidate in early adulthood [11], strengthening the case for prevention.

Against this burden, there is growing consensus that health services should account for masculine identities to improve men’s engagement and outcomes [12,13]. Physical activity (PA)—particularly resistance training (RT)—is a plausible, gender-congruent entry point that aligns with valued attributes (autonomy, strength, mastery) while delivering psychological and physical benefits, including mental health gains [14,15,16,17]. Meta-analysis shows that RT produces robust increases in muscular strength and hypertrophy across a range of loading schemes, supporting its utility as a cornerstone modality [18]. RT also yields cardiometabolic and body composition benefits, including meaningful reductions in resting blood pressure and decreases in fat mass [19]. Beyond physical effects, RT reduces depressive symptoms with a moderate pooled effect that is not contingent on improvements in strength [20,21], and it lowers anxiety symptoms in adults [22], with effects not moderated by sex—supporting applicability to men [23]. Reviews suggest that regular exercise increases brain-derived neurotrophic factor and supports hippocampal neurogenesis, modulates prefrontal–limbic circuits and hypothalamic–pituitary–adrenal axis activity, reduces pro-inflammatory cytokines, and influences monoamine signaling, which together may enhance stress resilience and mood regulation [24,25]. Yet, despite these benefits, RT remains underused at the population level: most adults fail to meet muscle-strengthening guidelines (≈10–30% compliance across countries), indicating a substantial participation gap [26]. Engagement disparities are shaped by education, self-efficacy, and program design—i.e., how RT is delivered matters [15]. Practical hurdles (e.g., equipment access, technical know-how) can be mitigated with clear instruction, accessible coaching, and body-weight options, but motivation-supportive delivery is essential. To convert initial resonance into sustained participation, programs should explicitly support autonomy, competence, and relatedness. In Self-Determination Theory (SDT) [27], support for these needs fosters need satisfaction and more autonomous motivation, which in turn predicts sustained behavior (e.g., adherence) and improvements in well-being—making SDT a strong fit for this population [27,28,29].

### 1.1. SDT

SDT [27,28] explains sustained health behavior through the satisfaction of three basic psychological needs: autonomy (a sense of volition and choice), competence (a sense of effectiveness and capability), and relatedness (a sense of belonging and mutual care). When these needs are supported, people internalize motives and act with more autonomous forms of motivation, which are consistently associated with stronger adherence and better well-being than controlled, pressure-based motives [27,28]. In physical activity, SDT constructs (i.e., autonomy, competence, and relatedness) predict both initiation and, critically, maintenance; interventions that engineer need-supportive climates reliably improve motivational quality and participation [29,30,31,32,33,34]. For young men, the social pathway may be especially important. Compared with women, men are less likely to seek help or disclose distress and are often under-represented in generic programs; gender-responsive approaches improve engagement [12,13,35]. Early signals that one belongs and can succeed—clear competence scaffolds and authentic opportunities to connect—are therefore pivotal to converting initial interest in RT into sustained participation [36]. In practical terms, effective RT onboarding should make belonging and mastery salient before training routines take hold. Guided by SDT, the present program therefore used both the NBI and RT components to support these three needs throughout the intervention.

### 1.2. NBI

A group-based format offers a direct mechanism for satisfying relatedness while also supporting competence and autonomy; programs that cultivate social cohesion, shared goals, and supportive norms produce better adherence and psychosocial outcomes than purely individual formats [37]. In the context of rising loneliness and social disconnection, embedding RT in cohesive groups normalizes participation and creates early mastery opportunities that consolidate competence [38]. Brief remote check-ins alone tend not to build strong early bonds; a short, in-person onboarding that seeds relatedness and competence before the RT phase is therefore advisable [39]. A brief, in-person nature-based intervention (NBI) can pragmatically deliver this onboarding by fostering early cohesion, shared identity, and mastery in a supportive setting [40]. Meta-analytic and review evidence links NBIs to reduced stress and improved mood, with additional signals for cognitive and cardiometabolic indices; in forest-bathing studies, short-term reductions in blood pressure and anxiety/depression are consistently observed [41,42]. These effects can be explained by multiple complementary theories. Specifically, attention restoration theory holds that natural settings engage effortless, softly fascinating stimuli, allowing directed attention to recover [43]. Stress recovery theory proposes that non-threatening natural cues trigger rapid parasympathetic down-regulation and emotional restoration [44]. The biophilia hypothesis posits an evolved tendency to affiliate with nature, which can affect regulation, meaning, and social connection [45]. Neuroimaging work indicates that brief nature exposure can reduce activation in regions implicated in rumination and stress, such as the subgenual prefrontal cortex and amygdala, and can produce short-term structural changes in hippocampal subfields, which are linked to stress-response inhibition and reduced rumination compared with urban walks [46]. Consistent with this, group-based NBIs are well positioned to prime early relatedness; evidence from group nature walks shows lower depression, reduced perceived stress, and improved well-being, and reviews indicate reductions in loneliness via belonging and cohesion [47]. Outdoor experiential programs also enhance self-efficacy in short timeframes [48], indicating that brief NBIs can deliver early competence cues before RT begins.

We realized that there is a gap at the intersection of an in-person nature-based onboarding, SDT-informed program design, and RT for young men. Recent syntheses of NBIs catalogue forest bathing, green exercise, horticulture therapy, and related formats, but do not report trials that integrate an NBI with other structured exercise programs [42,49]. Reviews of SDT-based physical-activity interventions describe autonomy-supportive coaching and need support delivered within program sessions, with limited description of structured pre-program onboarding phases to establish relatedness and competence before training begins [50]. Likewise, RT trials—especially those emphasizing physiological or mental health outcomes in young adults—rarely detail need-support strategies or group-cohesion protocols as part of their design [51]. Thus, within an SDT framework, the brief NBI served as a group-based onboarding to seed relatedness, competence, and autonomy before men transitioned into the RT phase, where these needs were further supported through choice of exercises, skill-building, and ongoing group check-ins.

### 1.3. Study Objectives

Guided by the ORBIT behavioral treatment development model (Obesity-Related Behavioral Intervention Trials) [52], which outlines a phased pathway from early intervention development (Phase I) through proof-of-concept studies conducted under optimized conditions (Phase IIa) and feasibility/pilot trials (Phase IIb) to larger efficacy and effectiveness research, this study aims to evaluate the proof of concept for a gender-sensitive program that used a brief, in-person nature-based onboarding to prime SDT needs, followed by a 6-week, RT program for young adult men. The primary objective was to determine whether the approach shows promise under optimized conditions, indexed by pre-specified benchmarks: satisfaction (NBI mean ≥ 3/4; RT mean ≥ 3.5/5); recruitment (≥42% of eligible prospects enrolled within the accrual window); retention (≥80% completion of post-intervention assessment); and an exploratory indicator of RT adherence (weekly check-in attendance; no a priori benchmark). These feasibility benchmarks (satisfaction ≥ 3/4 and ≥3.5/5; recruitment ≥ 42%; retention ≥ 80%) were specified a priori by the investigator team, in line with pilot/feasibility guidance recommending that studies define study-specific success criteria for recruitment, retention, and acceptability, and with recent early-phase behavioral trials that have used similar thresholds for satisfaction and retention [53,54,55]. To contextualize feasibility and illuminate perceived mechanisms of action, we conducted brief semi-structured exit interviews to assess acceptability (e.g., affective attitude, burden, perceived effectiveness). Secondary objectives were to explore preliminary signals on mental health indices. We expected that under optimized conditions, the dual-component NBI + RT program would meet these pre-specified feasibility benchmarks (satisfaction, recruitment, and retention) and that exploratory analyses would show reductions in depressive and anxiety symptoms.

## 2. Methods

### 2.1. Design

This single-group proof-of-concept study was guided by the ORBIT framework [52] and reported in accordance with CONSORT guidance for pilot/feasibility research [53]. In line with the ORBIT framework and CONSORT guidance for pilot and feasibility research, no formal a priori power calculation was undertaken. Instead, we planned a small cohort and focused on assessing feasibility, acceptability, and estimating within-participant change to inform the design and sample-size planning of a future, adequately powered randomized trial [52,53]. The program was delivered to intact cohorts in two sequential parts: a two-day, in-person nature-based onboarding (NBI) followed by a six-week RT phase supported by weekly videoconference check-ins. The planned cohort duration was 44 days (two days of NBI followed by six weeks of RT). Ethical approval was granted by the institutional Human Research Ethics Board of the University of Victoria (#22-0513).

### 2.2. Participants

Eligible participants were men aged 18–34 not meeting RT recommendations (i.e., <2 sessions/week). We defined ‘young men’ as 18–34 to capture emerging and early adulthood, a life stage in which most mental disorders first appear and suicide is a leading cause of death among youth and young adults, with public health agencies commonly classifying 15–34-year-olds within this ‘youth/young adult’ bracket, e.g., [56]. Recruitment ran from January to April 2023 using social media and posters placed in community centers, educational institutions, and job sites. Interested candidates contacted the study coordinator and completed screening, which included a recruitment letter, the Physical Activity Readiness Questionnaire PAR-Q+ [57], and a brief (~15 min) interview to confirm eligibility and availability. Those eligible provided written informed consent prior to enrollment.

### 2.3. Procedures

After consent, participants completed the baseline assessment within seven days prior to the NBI, which was delivered over two consecutive days on forested trail routes within the 565-acre Royal Roads University campus in Colwood, British Columbia. Sessions took place in an old-growth coastal forest bordering the Esquimalt Lagoon, using looped trails and small clearings for group discussions and activities. The program was delivered entirely in green space, with only brief views of the adjacent lagoon from some vantage points; nearby Hatley Castle, the formal gardens, and campus buildings were not part of the NBI, and the only buildings visible to participants were residential homes and an elementary school outside the fenced property when entering and exiting the site. Immediately after the NBI, participants completed a satisfaction measure. The six-week RT phase commenced thereafter, with participants following the program, attending weekly 1.5 h videoconference check-ins, and submitting weekly workout logs to document adherence and barriers. Post-intervention assessments (including RT satisfaction) were completed in the final three days of Week 6, and a brief semi-structured exit interview was conducted within seven days to assess acceptability and perceived mechanisms. The program was offered free of charge; no participation fees or monetary compensation was provided. All intervention sessions and group check-ins were facilitated by the second author, a graduate student in Child and Youth Care with over 10 years of professional outdoor education/leadership experience, counseling-practicum training, and multiple wilderness safety certifications. The assessment schedule (baseline; immediate post-NBI; weekly check-ins/logs; post-intervention; exit interview) is summarized in Table A1.

### 2.4. Intervention Materials Nature-Based Onboarding

The NBI was delivered outdoors across two consecutive days in four structured blocks per day. Activities were pre-mapped to SDT needs [28] and to the Motivational Behavior Change Techniques (MBCT) taxonomy, a classification of 21 SDT-consistent intervention techniques, each linked to support for autonomy, competence, or relatedness [58]. Mapping the program agendas to MBCT categories ensured that sessions systematically embedded need-supportive strategies (for example, providing meaningful choice, collaborative planning, and opportunities for group connection) throughout the program. To foster relatedness, the days opened with introductions and ice breakers, a facilitated men’s issues discussion (barriers to help-seeking, health and longevity), and recurring circle debriefs—an approach consistent with evidence that small-group identity/cohesion supports engagement [59]. To build autonomy, participants were given voice and choice during discussions and reflective tasks (e.g., a quiet “sit spot” and a nature-sculpture exercise using found natural items) that encouraged personal meaning-making in nature; structured nature contact is associated with affect regulation and well-being [60]. To support competence, the program included a guided nature walk with sensory awareness, structured team challenges requiring cooperative problem-solving, and an accessible psychoeducation block on stress regulation (autonomic nervous system/polyvagal states; comfort/challenge/danger zones) with a brief stepping-into-zones activity and practical anxiety management strategies [61]. Day 2 closed with a Men and RT Q&A that introduced the forthcoming RT phase and initiated individualized planning. Detailed intervention materials of the NBI are presented in Table 1.

### 2.5. Resistance Training Phase

The RT phase was delivered remotely over six consecutive weeks, with a minimum of two full-body sessions per week, each targeting the major muscle groups; compound lifts (e.g., chest press, squat, row, lunge, shoulder press, pulldown) were paired with body weight or band alternatives to ensure access outside a gym. Participants received an end-of-NBI demonstration, a video guide, and were encouraged to work out in pairs; progress was tracked on a weekly RT workout log (Table A2) recording sets, reps, and load to guide progressive overload and coach feedback. A 1.5 h evening group Zoom check-in was held once per week during the six-week RT phase, using a brief prompt set to provide troubleshooting and community support. The design features were also mapped to SDT: competence via instruction, clear exercise menus, and structured logging; autonomy via modality/location choices (weights, bands, or body weight; gym or home) and flexible scheduling; and relatedness via the small-group check-ins and optional partners—conditions linked to better exercise engagement and maintenance [33]. The RT program materials are presented in Table 2. The two-day NBI onboarding deliberately fed into this phase by seeding skills and motivation for RT, while the weekly human contact functioned like low-dose supervision, a strategy associated with higher adherence [62,63].

### 2.6. Measures

A brief self-report checklist at baseline captured age; education; marital status; employment status; annual household income; family role; and visible-minority status. Baseline questionnaires, including the demographic checklist, Patient Health Quesionnaire-9 (PHQ-9), and Generalized Anxiety Disorder-7 (GAD-7), were self-administered individually after enrolment and completed prior to the first group meeting. Participants received the forms by email and completed them at a time and place of their choosing; most returned the completed questionnaires digitally by email, and two participants completed paper copies and handed them in at the start of the NBI. Post-intervention PHQ-9 and GAD-7 were again self-administered individually after completion of the RT phase and returned digitally by email.

### 2.7. Primary Outcomes

#### 2.7.1. Satisfaction, Recruitment, Retention and RT Adherence 

Satisfaction for the 2-day NBI component was measured with a 7-item questionnaire on a 4-point Likert scale (1–4); sample items included “I was satisfied with the two days of nature time”, “I would do this again if I had the opportunity”, and “I feel prepared for the RT program ahead”. Satisfaction for the 6-week RT component was measured with a 9-item questionnaire on a 5-point Likert scale (1–5); sample items included “I am satisfied with the RT program I just completed”, “Having a group of men to check in with increased your motivation”, and “RT is now a higher priority in my life”. For both components, negatively worded items were reverse-coded, and a composite mean score was computed so that higher values indicate greater satisfaction. The recruitment rate was calculated by dividing the number of eligible men who enrolled during the accrual window by the number of eligible men approached. Retention was calculated by dividing the number of enrolled participants who completed the post-intervention questionnaire and exit interview by the number enrolled. RT adherence was indexed as the proportion of scheduled weekly check-ins attended; missed check-ins triggered individual follow-up to verify RT progress.

#### 2.7.2. Acceptability

At post-intervention, semi-structured exit interviews were used to contextualize the quantitative outcomes and assess acceptability and SDT-relevant processes (autonomy, competence, relatedness). The guide covered perceived impact and enjoyment of the program, accessibility/logistics, motivation to continue RT, confidence to perform RT independently, and changes in social connection around RT. Exit interview questions included, “What was the most impactful aspect of the program and why?”, “How enjoyable was it?”, “Was it accessible?”, “How has the intervention affected your level of motivation to participate in regular RT?”, “How competent do you feel in engaging in RT on your own going forward?”, “How has your ability to relate to other people who engage in RT changed?”, and “Did you find yourself motivated by this intervention? If so, please describe when you felt this motivation, how it affected you, and why you believe you became motivated”. Responses were documented and used to interpret acceptability and perceived mechanisms alongside the satisfaction measures.

### 2.8. Secondary Outcomes

#### Mental Health

Depressive symptoms were assessed with the 9-item PHQ-9 [64]. Participants rated each statement (e.g., “Little interest or pleasure in doing things”) on a 4-point Likert scale (0 = Not at all; 3 = Nearly every day). Anxiety was measured using 7-item GAD-7 [65]. The items (e.g., “Feeling nervous, anxious, or on edge”) were rated on a 4-point Likert scale from 0 (Not at all) to 3 (Nearly every day). In the present study, the Cronbach’s alpha coefficients of the PHQ-9 and GAD-7 were 0.75 and 0.85, respectively.

### 2.9. Data Analysis

#### 2.9.1. Primary Outcomes

- Satisfaction, Recruitment, Retention and RT Adherence

Satisfaction was summarized as composite mean scores (*SD*) for the NBI and RT satisfaction questionnaires and judged against a priori thresholds (NBI ≥ 3.0/4; RT ≥ 3.5/5). Recruitment was calculated as the proportion of eligible prospects who enrolled, and retention as the proportion of enrolled participants who completed the post-intervention assessment. Pre-specified progression criteria were recruitment ≥ 42% and retention ≥ 80%. RT adherence was summarized as the percentage of scheduled weekly group check-ins completed per participant, with missed contacts recorded and followed by one-to-one outreach; we report both the overall percentage and the distribution across participants. No benchmark was specified a priori for RT adherence.

- Acceptability

Post-intervention, semi-structured exit interviews were audio-recorded and analyzed in Microsoft Excel to contextualize the quantitative findings and assess acceptability and perceived mechanisms. Using an inductive, descriptive thematic analysis [66], the research team familiarized themselves with notes/transcripts, generated semantic codes aligned to feasibility and acceptability (e.g., enjoyment, burden, fit, perceived mechanisms), collated codes into candidate themes, and refined themes for coherence and distinctness. Qualitative coding and theme development were led by the second author, with the first author reviewing codes and themes. Both have training in qualitative methods and disciplinary backgrounds in behavioural medicine, and coding decisions were discussed with the wider author team. Representative quotations were selected to illustrate each theme. Qualitative results were integrated with the satisfaction scores to contextualize acceptability (i.e., explain what participants valued, what felt burdensome, and how the NBI helped or did not help early relatedness/competence for RT).

#### 2.9.2. Secondary Outcome

Within-participant change from baseline to post-intervention on mental health measures was described with pre/post means (*SD*) and mean change (Δ). Cohen’s *d* for dependent samples was calculated as the mean of the paired differences divided by the *SD* of those differences; negative values indicate improvement (lower symptom scores), and we interpret magnitudes using the usual small (0.20), medium (0.50), and large (0.80) guidelines [67]. Given the small single-arm proof-of-concept sample, we did not conduct formal hypothesis tests (*p*-values) for these outcomes.

## 3. Results

### 3.1. Sample Characteristics

All eight participants were male and aged 26–34 years, with most in the 30–34 category (5/8). Education ranged from some high school to graduate degrees; four held a college or graduate degree and three reported vocational/some colleges. Most were single (5/8), with three in long-term relationships (one married, two common-law). Employment was primarily part-time (4/8) or full-time (2/8), household income was polarized (3/8 < $20,000 vs. 2/8 ≥ $100,000), one participant was a parent, and one identified as a visible minority (see Table 3). No adverse events or safety incidents were reported.

### 3.2. Primary Outcomes

#### 3.2.1. Satisfaction, Recruitment, Retention and RT Adherence

For the NBI, the composite score was M = 3.4, *SD* = 0.8 on a 4-point scale, exceeding the ≥3.0 criterion. For the RT program, the mean score of the satisfaction scale was 4.3 (*SD* = 1.0) out of 5, above the ≥3.5 criterion (Table 4). Item-level summaries are shown in Table 5 and Table 6: the highest-rated NBI component was “Being in nature was an important factor in the experience” (M = 3.9, *SD* = 0.3), whereas “It was challenging to connect with others in the group” was lowest (M = 2.5, *SD* = 1.0). For RT, perceived impact (“RT made a positive impact on your life over the last six weeks”) and likelihood of continuing (“I am likely to continue an RT practice after this”) were high (both M = 4.8, *SD* = 0.7), while the scheduling burden (“Having RT in my schedule made my life more challenging”) was lowest (M = 3.3, *SD* = 1.3). Recruitment was 46% and retention was 100%, both above the a priori milestones of ≥42% and ≥80%, respectively (Table 4). RT adherence, indexed by attendance at six weekly check-ins, was 43/48 contacts (89.6%): 6/8 participants attended all six check-ins, 1/8 attended five, and 1/8 attended two. Missed check-ins were followed up individually to verify ongoing RT participation, and all eight completed the program.

#### 3.2.2. Acceptability (Qualitative Feedback)

Exit interviews (*N* = 8) indicated strong acceptability of the intervention and clarified how it was experienced, including enjoyability, accessibility, effects on RT motivation, perceived competence, and relatedness. Three themes were identified (Table 7). First, *satisfaction with time in nature* reflected benefits such as guided facilitation, the NBI curriculum’s educational value, group accountability, and a deeper connection with nature (1a); reported barriers were time and location, which participants described as manageable (1b). Second, *satisfaction with RT program* emphasized increased motivation and competence for RT alongside mental health benefits (e.g., stress/mood; 2a), while time demands and motivational dips were noted but did not prevent progress (2b). Finally, *feedback on format and motivational changes*—participants valued the NBI’s role in program buy-in and group trust (3a), emphasized weekly check-ins for accountability and guidance (3b), and reported gains in autonomy and competency around RT for future application (3c). Overall, the qualitative findings triangulate the questionnaire results by explaining why participants were satisfied and how the NBI and weekly check-ins supported motivation and skill development for sustained RT.

### 3.3. Secondary Outcomes

#### Mental Health

Scores on PHQ-9 and GAD-7 improved from pre- to post-program. In particular, PHQ-9 total scores declined from 12.33 to 6.48 (Δ = −5.85), with 7/8 participants showing within-person improvement; the mean individual percent change was −42.25%. This reflects an average shift from moderate (10–14) at baseline to mild (5–9) post-program. GAD-7 total scores declined from 11.41 to 5.67 (Δ = −5.74), with improvement in 8/8 participants; the mean individual percent change was −50.71%, likewise an average shift from moderate (10–14) to mild (5–9). Standardized mean changes were large (PHQ-9: |*d|* = 1.44; GAD-7: |*d|* = 2.48) (see Table 8).

## 4. Discussion

The aim of this proof-of-concept study, framed within SDT [27], was to assess the potential benefits of a consecutive dual intervention consisting of an in-person NBI and a subsequent virtually supported RT program for male physical and mental health and well-being. The rationale stems from the disproportionate social struggles of young men [3], and all-cause mortality [1]. Under pre-specified progression criteria, the trial met recruitment and retention benchmarks, achieved high satisfaction with both components, and showed reductions in depressive and anxiety symptoms alongside strengthened motivation to engage in RT—together indicating promise for this SDT-informed approach to male mental health and well-being.

### 4.1. Primary Outcomes

Acceptability and satisfaction were strong across both components. Specifically, NBI satisfaction exceeded the a priori threshold (M = 3.4/4). Exit interview narratives clarified the drivers: participants highlighted the *guided learning* and *educational value* of the outdoor curriculum, *accountability* within the small male group, and a heightened *sense of connection to nature*. This aligns with evidence that structured nature contact supports affect regulation and well-being and that benefits increase once weekly exposure approaches ~120 min—plausibly achieved by our two-day format [41,60]. Interpreted through SDT, the guided activities and skill practice likely supported autonomy and competence, while the group context fostered relatedness [28]; satisfaction of these needs is reliably associated with higher program satisfaction and sustained PA engagement [30,33]. Participants also noted that starting together in person helped build trust and buy-in, but the two-day dose posed scheduling barriers for some; a shorter or virtual option could increase access if cohesion is preserved in other ways. Further, the relatively lower “connecting with others” item (M = 2.5) reflected one reported interpersonal mismatch in interviews, suggesting deliberate early cohesion-building and attention to group composition in the next iteration [59,68].

Satisfaction with the RT phase was high (M = 4.3/5) and adherence was strong, with 6/8 attending all six check-ins. These findings are consistent with evidence that regular human contact—even brief, structured touchpoints—provides accountability and social support (e.g., weekly “check-ins” or supervision) that raise exercise participation and adherence [69]. It also fits SDT: the check-ins likely supported competence (feedback, troubleshooting) and relatedness (feeling noticed/connected), both of which predict sustained exercise engagement [30,33]. The program also emphasized concrete planning and scheduling—individualized RT plans (exercises, sets/reps), weekly training schedules, and workout tracking—which supported adherence between check-ins [70]. The item about maintaining motivation without the group scored second lowest, however, suggesting the importance of relatedness and graduated support. The lowest-rated item—“Having RT in my schedule made my life more challenging”—points to some perceived time/scheduling burden, a ubiquitous barrier to exercise [30]. Yet, two design features appear to have mitigated these burdens: (i) remote, brief check-ins, which preserved human support without added travel time, and (ii) supervision/accountability, which improves session attendance and progression relative to unsupervised programs [63,71]. Several participants suggested an optional mid-point or end-of-program in-person meet-up to recalibrate plans and reconnect; future trials should test this brief booster to sustain engagement in longer, larger randomized designs.

Retention was 100% (8/8 completed), which is notable given the commitment (a two-day in-person NBI followed by six weeks of RT). That result is plausible given this study’s in-person onboarding during the NBI, which established rapport and clear expectations before the remote phase—a feature repeatedly associated with better engagement in blended (face-to-face + eHealth) programs [39]. In addition, our pre-scheduled weekly contacts and proactive follow-up after any missed contact align with trial-retention evidence: systematic reviews show that timely reminders and brief telephone outreach improve participant retention and reduce loss to follow-up [72]. Finally, beginning in a small, male-only group likely helped early cohesion/identification, which is linked to staying power in group-based PA programs—another plausible contributor to full completion here [73].

The study achieved a 46% recruitment rate over just under four months—above the a priori ≥42% milestone—using a mix of direct contact/referrals plus social media and posters. Recruiting men to health and mental health studies is consistently difficult due to stigma, low help-seeking, and limited identification with conventional services [3,13]; our yield is therefore competitive for early-phase work. Reviews of male recruitment highlight exactly these barriers and recommend gender-sensitized framing, trusted settings, and peer/organizational referrals—tactics we used and should amplify in the next phase [74]. Evidence also shows that social media can cost-effectively boost enrollment in health and mental health research; targeted campaigns (e.g., age-banded Facebook/Instagram ads) typically improve reach and reduce cost per enrollee versus traditional methods [75]. For stronger generalizability and speed, we recommend partnering with male-identified community venues (e.g., sports clubs) and using male-friendly, action-oriented messaging—approaches that have driven high male engagement in programs like Football Fans in Training [76] and related club-based initiatives [77]. Together, the acceptability, satisfaction, recruitment, and retention results provide a clear *signal of promise* for this SDT-informed, NBI onboarding to virtual RT sequence and identify actionable refinements (early cohesion-building; flexible scheduling; lighter-touch back-ups when live attendance is difficult). Under ORBIT, these Phase IIa signals justify advancing to a Phase IIb feasibility/pilot to optimize procedures and dosing before any efficacy testing.

### 4.2. Secondary Outcomes

Across the eight participants, depressive and anxiety symptoms decreased on both measures from pre- to post-intervention, consistent with movement from the moderate to mild symptom ranges on each scale [64,65]. We believe that this combined NBI and RT sequence helps for complementary reasons. First, the NBI plausibly targeted cognitive–affective processes linked to mood and anxiety [78]. Experimental and review evidence shows that time in natural settings can reduce maladaptive rumination, while also restoring directed attention (Attention Restoration Theory) [43] and supporting stress regulation [41,44,60,78]. Conceptual syntheses likewise outline multiple pathways through which nature experience supports mental health (affect regulation, cognitive restoration, social connection), aligning with participants’ reports of educational value, accountability, and feeling more connected to nature [60].

Second, the RT phase is supported by meta-analytic evidence that RT reduces depressive symptoms with moderate effects and also improves anxiety—across ages and health statuses [20,22]. We also speculated that the RT program was able to promote mastery and self-efficacy, social support/encouragement from regular check-ins, and the stabilizing effects of a routine that also tends to improve sleep—all factors tied to better mood and anxiety management in exercise research [79,80,81]. In our design, the NBI likely boosted the three psychological needs: autonomy/competence/relatedness [27], easing the transition into RT; in turn, the structured, coached RT with weekly touchpoints supported skill mastery and adherence—factors repeatedly associated with stronger symptom improvements in exercise trials [82].

In addition to these psychological and behavioral processes, reviews [24,46,60] indicate that regular exercise and brief nature exposure can influence neuroplasticity and stress regulation systems, offering a further plausible pathway for mood and anxiety improvements, although we did not assess these mechanisms directly in this study. Taken together, these complementary cognitive–affective, behavioral, and biophysiological pathways provide a coherent explanation for the observed reductions in depressive and anxiety symptoms. Yet, the findings should be interpreted cautiously given the small, uncontrolled sample.

### 4.3. Limitations and Future Directions

This study had several limitations that need to be acknowledged and addressed in future research. First, as a proof-of-concept trial, limitations include a small sample that may not generalize to broader populations. Future studies should track external influences (e.g., via weekly journals), increase sample size, and use a comparison condition. Second, we intentionally focused on self-selected young men within a narrow age band (mid-20s to early-30s) to create a relatively homogeneous, high-risk cohort for this initial feasibility test; as a result, findings should be interpreted primarily for young adult men and may not generalize to older men, younger adolescents, or women. To enhance generalizability, future trials should broaden reach via stratified recruitment (community clubs/gyms, workplaces, campus services), targeted ads by age and socioeconomic status, and tracked refusals to quantify reach. Third, group dynamics mattered: one interpersonal mismatch dampened “connection” ratings and reduced a participant’s group attendance. In future trials, this could be mitigated with a brief pre-group orientation and ground rules, early cohesion exercises, and a contingency plan (facilitator mediation or re-assignment). Finally, while the two-day, in-person NBI appeared to build early buy-in, it also created scheduling barriers for some participants. Future trials should compare condensed formats (one day or two half-days) and a guided virtual option, incorporating an early cohesion-building task to preserve the social benefits.

## 5. Conclusions

This proof-of-concept study, grounded in self-determination theory, tested a consecutive program that began with a brief in-person nature-based intervention and transitioned to a virtually supported RT phase for young men. Feasibility signals were positive: recruitment reached 46%, retention was 100%, and mean satisfaction exceeded pre-specified thresholds for both components (NBI 3.4/4; RT 4.3/5). In this small, single-arm, non-clinical sample, PHQ-9 and GAD-7 scores decreased from moderate to mild ranges, indicating improvements in symptomatology rather than incidence or remission of depressive or anxiety disorders, and qualitative feedback highlighted gains in motivation, confidence, and connection. The combined format is innovative in pairing an in-person NBI onboarding—to seed autonomy, competence, and relatedness—with a remote, coached RT routine that maintains support while reducing burden. Building on these signals, the appropriate next step in the ORBIT pathway is a Phase IIb feasibility/pilot trial with a randomized comparison group and longer follow-ups.

## Figures and Tables

**Table 1:** Two-day NBI materials.

Day/Block	Activity	Core Content	Intended Function/Process	SDT Needs Targeted	MBCT *
Day 1— 09:00–10:15	Introductions and ice breakers	Welcome, agenda, name/game ice breakers	Establish familiarity, psychological safety, initial cohesion	Autonomy Competence Relatedness	MBCT1, MBCT3, MBCT16
Day 1— 10:15–10:45	Break				
Day 1— 10:45–12:00	Men’s issues group discussion	Guided discussion on help-seeking, health, purpose of program	Shared norms, meaning/values clarification, buy-in	Autonomy Competence Relatedness	MBCT2, MBCT3, MBCT4, MBCT11, MBCT16
Day 1— 12:00–13:00	Lunch				
Day 1— 13:00–14:15	Physical activity in nature	Quiet nature walk; team challenge/problem-solving	Connection, attentional reset, cooperative efficacy	Autonomy Relatedness	MBCT10
Day 1— 14:15–14:45	Break				
Day 1— 14:45–16:00	Nature connection/debrief	Nature sculpture; solo “sit spot”; circle debrief	Personal meaning-making, affect regulation, nature connection	Autonomy Relatedness	MBCT1, MBCT3, MBCT10, MBCT12
Day 2— 09:00–10:15	Ice breaker and reflection	Weather check-in; “silent predator–protector” group activity; recall task	Re-energize group, reinforce cohesion	Autonomy Competence Relatedness	MBCT3, MBCT10, MBCT11, MBCT16
Day 2— 10:15–10:45	Break				
Day 2— 10:45–12:00	Psychoeducation	Autonomic nervous system; polyvagal overview; comfort/challenge/danger zones; stepping-into-zones activity; coping strategies	Stress literacy, anxiety management skills	Autonomy Competence Relatedness	MBCT4, MBCT10, MBCT12, MBCT15, MBCT16
Day 2— 12:00–13:00	Lunch				
Day 2— 13:00–14:15	Team-building	Rope/bucket lift; “river crossing” foam-pad challenge	Trust, communication, problem-solving under challenge	Competence Relatedness	MBCT4, MBCT8, MBCT10, MBCT12, MBCT16
Day 2— 14:15–14:45	Break				
Day 2— 14:45–16:00	Men and RT Q&A/close	Benefits of RT; exercise demos; how to track; craft individualized RT plan	Readiness for RT, clear next steps and expectations	Autonomy Competence Relatedness	MBCT4, MBCT5, MBCT6, MBCT10, MBCT16

Note. MBCT = motivational behavior technique; * Numbers correspond to the MBCT Classification as defined by Teixeira et al. (2020) [58].

**Table 2:** Six-week RT program materials.

Component	Brief Description/Dose	Intended Function/Process	SDT Needs Targeted
Transition from NBI	RT plan crafted at end of Day 2 (exercises, sets/reps, schedule, tracking)	Clear goals, immediate next steps, continuity from NBI	Autonomy Competence
Exercise prescription	Minimum 2 full-body sessions/week (aim ≈ 90 min/week via 2 × 45 or 3 × 30); major muscle groups each session	Clear dose target, flexibility of scheduling/location	Autonomy Competence
Exercise menu and alternatives	Compound lifts (chest press, squat, row, lunge, shoulder press, pulldown) with body-weight/band options	Choice + accessibility; progressive overload possible regardless of equipment	Autonomy Competence
Instructional support	End-of-NBI demos + video guide to exercises and safety cues	Skill acquisition, perceived competence	Competence
Self-monitoring and feedback	Weekly RT workout log (sets, reps, load) to track progress	Progressive overload; visible progress reinforces mastery	Autonomy Competence
Weekly group check-in	~1.5 h Zoom (prompts: wins/challenges, 1–10 weekly rating, Q&A, plan for next week)	Accountability, troubleshooting, encouragement	Competence Relatedness
Partner option	Encouraged to work out in pairs when feasible	Social support outside sessions	Relatedness
Follow-up for missed check-ins	Individual outreach to confirm training and re-engage	Lapse recovery, maintain engagement	Competence Relatedness

**Table 3:** Participants’ demographic information.

Demographic Profile	Frequency (*N*)	Percentage (%)
Age in Years		
26–29	3	37.5
30–34	5	62.5
Education		
Some High School	1	12.5
Vocational School or Some College	3	37.5
College Degree	3	37.5
Professional or Graduate Degree	1	12.5
Marital Status		
Single	5	62.5
Married	1	12.5
Common Law	2	25.0
Employment		
Full-time Employment	2	25.0
Part-time Employment	4	50.0
Temporarily Unemployed	1	12.5
Others	1	12.5
Annual Family Income		
Less than $20,000	3	37.5
$20,001 to $40,000	2	25
$40,001 to $75,000	1	12.5
$100,000 or Above	2	25
Family Role		
Parent	1	12.5
Independent	7	87.5
Visible Minority		
Yes	1	12.5
No	7	87.5

**Table 4:** Participant intervention feasibility and acceptability success criteria.

Outcome	Success Criteria		Results	
	NBI	RT Program	NBI	RT Program
Satisfaction	Mean score ≥ 3 points	Mean score ≥ 3.5 points	Mean score = 3.4 points	Mean score = 4.3 points
Recruitment Rate	≥42%		46%	
Retention Rate	≥80%		100%	

**Table 5:** Participant (*N* = 8) satisfaction with the NBI.

Item	M ± SD
NBI components	
I was satisfied with the two days of nature time	3.6 ± 0.5
I would do this again if I had the opportunity	3.4 ± 0.9
Having only males in the group was a benefit	3.4 ± 0.7
It was challenging to connect with others in the group	2.5 ± 1.0
Being in nature was an important factor in the experience	3.9 ± 0.3
I am likely to integrate this experience in my life	3.5 ± 0.5
I feel prepared for the RT program ahead	3.4 ± 1.0
Overall	3.4 ± 0.8

**Table 6:** Participant (*N* = 8) satisfaction with RT program.

Item	M ± SD
RT program	
I am satisfied with the RT program I just completed	4.5 ± 0.7
RT made a positive impact on your life over the last six weeks	4.8 ± 0.7
I am likely to continue an RT practice after this	4.8 ± 0.7
Having a group of men to check in with increased my motivation	4.6 ± 0.5
I am likely to maintain motivation without other people involved in the same program	3.5 ± 1.0
My mental health was positively affected by the inclusion of RT in my week	4.5 ± 0.7
Having RT in my schedule made my life more challenging *	3.3 ± 1.3
I often considered quitting the program *	4.4 ± 1.0
RT is now a higher priority in my life	4.3 ± 0.7
Overall	4.3 ± 1.0

Note. * Negatively worded items were reverse-scored so that higher values reflect greater satisfaction.

**Table 7:** Themes, subthemes, and selected quotes supporting the themes.

Themes	Subthemes	Illustrative Quotes
1. Satisfaction with *Time in Nature*	1a. Enjoyment and benefits of the intervention	“The leader, the person responsible for facilitating the experience, gave me some insightful thoughts that changed my perspective on some things.”—Erik “That initial launching platform with nature and nature connection with the other guys, yourself and myself actually having a real relationship.”—Ryan
	1b. Challenges to participation	“Making the time to take two days was challenging at first but once I committed, it was not a problem and worth the effort.”—Ned “The biggest obstacle was getting there but you did pick a place that was pretty accessible by public transit.”—Dan
2. Satisfaction with RT Program	2a. Enjoyment and benefits of the intervention	I’ve noticed better muscle firmness. And when I’m lifting, I’m not thinking about too much else. I’ve felt a natural desire to do it and it alleviates stress.”—Joe “I didn’t used to enjoy lifting because I felt for me it was kind of boring, but now, I realized that it was because I was not pushing myself hard enough. I was like kind of afraid of hurting myself. So, I didn’t really put a lot of weight on or anything. Also, because I didn’t do a lot of effort, I didn’t see a lot of changes. So that was kind of discouraging, too. And now, yeah, I always like trying to push myself a little further.”—Tal “I think it’s led to 180 degrees shift—I went from not caring to, if anything, I’m going to keep up the same routine.”—Ryan
	2b. Challenges to participation	“The time it takes, and my other responsibilities are a challenge right now.”—Tal “My original motivation is not there—longevity—I noticed this when I miss one workout because I was away, but I did exercise. I felt a bit like I failed but as soon as I got back, I went instantly.”—Sal “I think that’s the hardest part is getting so you feel comfortable to keep going and keep learning.”—Dan
3. Feedback on Intervention Format and Motivational Changes	3a. NBI value for RT	“The time in the forest was the most impactful by far.”—Erik “Maybe a second day of nature time in the middle or something. I think I would have gotten more enjoyment out of that, because I really love being out in nature and physically being around other people doing things like this together.”—Joe “There were a bunch of things that made that it possible. I think a big part of that… would be the confidence I had that I wasn’t just picking up “[BS]” on the Internet. I’m being given some structure and a plan by someone who knows something and is giving me a good base starting off point and paired with the accountability. And there’s just so many layers the accountability of meeting in person, and I think nature is 100% the right place for that. And then the check-ins.”—Ryan
	3b. Weekly check-ins and support	“It was a really good time talking with all of you. I felt like we had a really unique good, strong bond, and that’s what has led me to work out so much in the past week, too.”—Erik “Having a meeting once a week helps with motivation.”—Joe “Just having someone with a lot of expertise, but not the kind that lays any hammers down and just being very accepting and accommodating of each person where they’re at. So that’s been very motivating.”—Dan
	3c. Applications going forward	“Motivated me to improve sleep and nutrition as well.”—Sal “I feel, way more competent that I felt 6 weeks ago. I feel that I still have a lot of things to learn. I would never even think about saying that I have the same knowledge or expertise than people who have been doing this for years, but at least I don’t go feeling that I don’t know what I’m doing.”—Tal “And now I kind of identify people in my existing social group that are interested in working out. And there’s these different weight training classes that I can get involved. And there’s a bunch of different stuff that’s out there, and that I wouldn’t have ventured into. It wasn’t for this.”—Ryan

Note. All participant names are pseudonyms to protect confidentiality.

**Table 8:** Preliminary PHQ-9 and GAD-7 (Pre-Post) participant outcomes of the NBI-RT (*N* = 8).

Outcome	Pre-Intervention (M)	Post-Intervention (M)	M_diff_	Improved (*N*)	Cohen’s *d*
PHQ-9	12.33	6.48	−5.85	7/8	−1.44
GAD-7	11.41	5.67	−5.74	8/8	−2.48

## Data Availability

The data presented in this study are available on request from the corresponding author. The data are not publicly available due to privacy and ethical restrictions imposed by the University of Victoria Human Research Ethics Board and the conditions of participant consent. De-identified quantitative data may be made available from the corresponding author on reasonable request, in line with these ethical and consent requirements.

## Abbreviations

NBI	Nature-based intervention
RT	Resistance training
PA	Physical activity
SDT	Self-determination theory
ORBIT	Obesity-Related Behavioral Intervention Trials (behavioral treatment development model)
MBCT	Motivation and Behaviour Change Techniques (taxonomy of motivation and behavior change techniques)
PHQ-9	Patient Health Questionnaire-9 (depressive symptoms scale)
GAD-7	Generalized Anxiety Disorder-7 (anxiety symptoms scale)
GBD	Global Burden of Disease
PAR-Q	Physical Activity Readiness Questionnaire
SD	Standard deviation
CONSORT	Consolidated Standards of Reporting Trials (reporting guideline for trials)

## Appendix A

**Table A1:** Full assessment schedule.

Data Collection Item	Schedule	Primary	Secondary
Prospect tally	Prior to start date	X	
PAR-Q and consent form	Prior to start date	X	
Recruitment rate	Start date—day 1	X	
Background questionnaire Sociodemographic RT history PA history Health-related questions	Start date—day 1		
PHQ-9 and GAD-7 (pre-intervention)	Start date—day 1		X
NBI satisfaction questionnaire (including SDT material)	End of NBI—day 2	X	
Workout log × 6	Weekly after NBI	X	
Group video conference call check-in questions	Weekly	X	X
PHQ-9 GAD-7 (post-intervention)	End of RT program Day 44		X
Exit interview (including SDT material)	End of RT program ~ Day 44		X
Retention	End of RT program ~ Day 44	X	

Note. X indicates that the data collection item contributed to the corresponding outcome category.

**Table A2:** RT workout log (week 1 sample).

	Week 1		Week 1	
	Session 1		Session 2	
	Sets/reps	Weight	Sets/reps	Weight
(Example)	Set 1:10 reps	30 kg (65 lbs)	Set 1:10 reps	65 lbs
	Set 2:10 reps	30 kg (65 lbs)	Set 2:8 reps	75 lbs
	Set 3: 8 reps	30 kg (65 lbs)	Set 3: 10 reps	65 lbs
Chest press				
Squat				
Row				
Lunge				
Shoulder press				
Pulldown				
